# Supplementary material for: Primary Structure and Coding Genes of Two Pheromones from the Antarctic Psychrophilic Ciliate, Euplotes focardii
Source: Microorganisms. 2022 May 25;10(6):1089. doi: 10.3390/microorganisms10061089 (PMC9229436; doi:10.3390/microorganisms10061089)
Supplement: Supplementary file 1 [file microorganisms-10-01089-s001.zip › microorganisms-1711669-supplementary.pdf]

Supporting Information

Primary structure and coding genes of two pheromones from the Antarctic psychrophilic ciliate, *Euplotes focardii*

Claudio Alimenti, Annalisa Candelori, Yaohan Jiang, Pierangelo Luporini and Adriana Vallesi*

Laboratory of Eukaryotic Microbiology and Animal Biology, School of Biosciences and Veterinary Medicine, University of Camerino, 62032 Camerino [MC], Italy;
claudio.alimenti@unicam.it; annalisa.candelori@unicam.it; yaohan.jiang@unicam.it;
piero.luporini@unicam.it; adriana.vallesi@unicam.it
*Correspondence: adriana.vallesi@unicam.it

Figure S1: Nucleotide sequence alignment of the *mac-ef-1* and *mac-ef-2* macronuclear pheromone coding genes (telomeric C4A4 and G4T4 repetitions at the 5′ and 3′ extremities are omitted). In each gene sequence, the open reading frame encoding the pheromone precursor is indicated by capital letters; putative GTA/TAG splicing sites and a TTATTT poly(A) signal are in bold and boxed, respectively; nucleotide sequence identities are highlighted in grey.

```
mac-ef-1    ggattgtattctcaaagttgagcctactttaatattaaatttactatataaatatggctaaagcatagaa 70
mac-ef-2    tgaacactctctcaaggttggagctatt------------------------------------------ 28

mac-ef-1    ttctaacatttattgtcaaattagctcgacattcgcaattctaaataaagcaccttctcttaaaagtatc 140
mac-ef-2    -------------------------------taaattttatttctaaaatgtcttcttgtaaaaatatc 67

mac-ef-1    taaactccctaaagacatgaattttgtaaatatattgttgagaaaggatgtgcttagtttttcaaagc 210
mac-ef-2    taaattccaattata---tgattttagtaaaagtactgtttataaatgaggaagtgcttaagttttaagt 134

mac-ef-1    actaaaccctcaaataaatattaagaagattcatcttcgtagtcatattcatgacaaagtgagtctgatc 280
mac-ef-2    acagaaccttcaaactt------ataaaaatcagctttatagctatttcatataaactaagattggta 198

mac-ef-1    catccatctctctacagtaagtacaacagttatatccgtagtttcc------------------------ 326
mac-ef-2    cttcaatctgccttccgtgcgagaaacaatttatctgaagttaggaatattatttattttaattattca 268

mac-ef-1    ------------------------------------------------------------------tcca 330
mac-ef-2    aagatttcttgaatatgtaaatcttttaaaccattgctctgttattaaattagctttgaagttgccacta 338

mac-ef-1    tacaagtaatcaacaggatcgttgttatttcccatgatgctggagcataaagtctgtgctgttgctagta 400
mac-ef-2    tccaagttatcaataggattattgttaacttccataatattataacataaaatatgtacagtttctact- 407

mac-ef-1    atttgtcacccttcttcaaaagaatatagtatatttatactgtgtaaatatatgaacaatatagataaaa 470
mac-ef-2    ---------------------------------------------------------------------- 407

mac-ef-1    atagattttattattgaccgtaccaactgatttacagtcaatacttccctgatgagtatct-tgacattc 539
mac-ef-2    ----------------------ataattagatgacagacaatatatccaatatgaatattctaacctctc 455

mac-ef-1    atcctcttctataacgtaatgattaggacttgaaacaataagcgacataatgtctttgatagatatttaa 609
mac-ef-2    atttgttcctgtaaggttataaaaaggacttgaaacaataggagagagtgttgtttgatcttttataat 525

mac-ef-1    atcatcctaagcaaaaaaatctg-----------------------------cgaaaattttga------ 644
mac-ef-2    cattctcaagccaacagaattggattagaaataatttattctcaaattcatattaatattttctaaaaat 595

mac-ef-1    gaagaataaaattatgctatttgctttttacctctaaggtttaaaaggcttgatgaactataagtggaag 714
mac-ef-2    tgttatccatgttacagttttgatttttactctaaagttaaaaaagctacgaaaatgataatggaagg 665

mac-ef-1    aagacttactgtatattccattttgtcaaactttcttccatcactgtctttctttcctgctctgaatcc 784
mac-ef-2    cagatttactagatactctatttctattcgtctttcttctattgttgtctcttctttatgctctgaatct 735
```

mac-ef-1 agaaaca**gtag**cga**gta**tttatcacacaa**gtagta**a**gta**taaagcggaattgaatatttggtcaatttttt 854
mac-ef-2 acagacagttgtga**gta**tccgtcatgcga**gtag**tccgt-------------------------------- 773

mac-ef-1 catttatgaagaaggagtgcttcatttgagatttgaataattccaaataagaatattattatttttaat 924
mac-ef-2 -- 773

mac-ef-1 gattttccattaataattcaaattaattatactcctttcattcggaggatctta**tagta**aagttc**gta**ta 994
mac-ef-2 ---atc 776

mac-ef-1 gagcaattttatgaaaaatatttctaattatgataaacttggtcgataatttaaaaatctactttac 1064
mac-ef-2 gaatttcatattatgaaattttcgaattatgatttgcgaaagtttcaatttggaaaattatttac 846

mac-ef-1 aaaatttgtgacaagttgctgatcgggagtctgtt**tag**cctgatcactgtcaaacttctgatcatcaattc 1134
mac-ef-2 aaagtttgtgacaagttgcttatcagaagtctgttcatctgatcactgt**tag**atttctgatcatcaattc 916

mac-ef-1 ata**tag**agaaa**tag**gcatatcct**gtag**atgacctctgatacggctcttttggtctttctcttttaattaa 1204
mac-ef-2 attctgttcggtcttttat--- 935

mac-ef-1 ctaaaatataagatcaactcgttggctaaaattgaatcctcttcac**tag**aaaagtgaaataatcaccaat 1274
mac-ef-2 -- 935

mac-ef-1 ataatat**gta**aatttgtgataaatatgtcgaaatgaattttaaatttaaggaatgcataccaataagaaa 1344
mac-ef-2 -----------------------cgtctaccacttacttttctcatttgatt**tag**cttaccatttaaaat 982

mac-ef-1 tgctgatgaactaaaaaggattaaataagct**tag**gc**tag**cctattgtcacctaaccaatcatctcctaaa 1414
mac-ef-2 atggaag----------gaaagaa**gta**aga-**tag**a--------------tcttattaataaccttctaaa 1027

mac-ef-1 **gta**cgagaagtcttaataaaaaaatgg**gtag**tgcatatttcttactacat-**tag**aaatacttcaatggca 1483
mac-ef-2 gcattcccat---ctttataaaagatgaaaatgaatatttcttaactatttaaaaatactaa---agaa 1091

mac-ef-1 catgagaatttcagaaggaaaattaatttatttaa**gta**atggaaaaattattacgctgaattcgataatt 1553
mac-ef-2 aatgagaaattttta------------------**tagta**-gcaaaacgctttaaatattattttatgctt 1142

mac-ef-1 ctaagtgaaaatttgat-ccaaata**tag**gcctatggctatccct**tag**gtt**tag**acaaggattttccaaa 1622
mac-ef-2 ttatgcta**tag**gaatattcttattatactct-**tag**atttgtgctgggatctttgcataagttttgtcta 1211

mac-ef-1 aagcctatacctctt**taggta**a**gtagta**tcagaaccatcagtctaacagataactgt**tag**cccgatcagc 1692
mac-ef-2 atttgaatttctatcaaagagaaatcatcagattcatcagttcaacagacaactgt**tag**cccgatcagc 1281

mac-ef-1 aacttgtcacaaacttt**gta**aa**gta**agatatttaaaaatcgaaattaatttatctacaaattaaaattta 1762
mac-ef-2 aacttgtcacaaacttt**gta**aa**gta**agatatttaaaaatcgaaattaatttatctacaaattaaaattta 1351

mac-ef-1 ttaatcATGAAAATCACTATCTTAATTGTAACTCTTTGTGTCCTCCTTGCTTCTGTACAAGGATTTAAAG 1832
mac-ef-2 ttaatcATGAAAATCACTATCTTAATTGTAACTCTTTGTGTCTTCCTTGCTTCTGTACAAGGATTTAGAG 1421

mac-ef-1 CTAGGCCTCATGCTAACGCTAATGCTAACGCTCAAGCTCAAGTCCAAACTAGCACTGGATCTGATTGCCA 1902
mac-ef-2 CTAAGGCTCATGCT------------------CAAGCTCAAGTCCAAACTAGCACTGGATCTGATTGCCA 1480

mac-ef-1 CGGAGATACTGAATACCTTATTGATGATGAGAGTTGCGGATGTGACCATAATGGACCAAGCACTTGTGAG 1972
mac-ef-2 CGGAGATACTGAATACCTTATTGATGATGAGAGTTGCGGATGTGACCATAATGGACCAAGCACTTGTGCG 1543

mac-ef-1 ACAGCGTATTCATTAGATCAATCTAATCATGTATTAAAGGATGCTTATATTGAAACTCTTTGTGGAACTG 2052
mac-ef-2 ATGGCGTATTCATTAGATCAATCTAATCATGTATTAAAGGATGCTTATATTGAAACTCTTTGTGGAACTG 1613

mac-ef-1 ACTATATGGGATCTGACGGTTTTGGTGATTACAATTATGGACTCGACTATGGATGTAACTGCTGTCAAGT 2132
mac-ef-2 ACTTTATGGGATCTGACGGTTTTGGTGATTACAATTATGGACTCGACTATGGATGTAACTGCTGTCAAGT 1683

mac-ef-1 TTGTATTGATTATGGACATTGCGGAACAGTTGAATAActcgtagtaatagaattatttattgagaagat 2182
mac-ef-2 TTGTATTGATTATGGACATTGCGGAACAGTTGAATAActcgtagtaatagaattatttattgagaagat 1753

mac-ef-1 acagaatcaggcaaagaattgatctgtaatttctccatcaaaacaaatttgattaactcgcttttattat 2252
mac-ef-2 acagaatcaggcaaagaattgatctgtaatttctccatcaaaacaaatttgattaactcgcttttattat 1823

mac-ef-1 gggtttttggtactttttattttattattagaggaatattctggttcaatagtgtataacttccat 2318
mac-ef-2 gggtttttggtactttttattttattattagaggaatattctggttcaatagtgtataa------- 1882

Figure S2: Amino acid sequence alignment of *E. focardii* pheromones E*f*-1 and E*f*-2 with other *Euplotes* pheromones carrying the $CX_{1\text{-}3}CCX_{2\text{-}3}C$ motif (boxed). The sequences E*c*-1, E*c*-2, E*c*-3 and E*c*-α are representative of the *E. crassus* pheromones, E*n*-1, E*n*-2 and E*n*-A1 of *E. nobilii* and E*p*-1 and E*p*-2 of *E. petzi.* Gaps are inserted to maximize the alignment; the positions of Cys residues are highlighted in grey.

```
Ef-1 SDCHGDTEYLIDDESC---GCDHNGPSTCETAYSLDQSNHVLKDAYIETLCGTDYMGSDGFGDYNYGLDYGC-N-CCQV-CIDY-GH----CGTVE
Ef-2 SDCHGDTEYLIDDESC---GCDHNGPSTCAMAYSLDQSNHVLKDAYIETLCGTDFMGSDGFGDYNYGLDYGC-N-CCQV-CIDY-GH----CGTVE
Ec-1  GC----------FGCAPTICQF-----CE-A---IVNPNPDVY------CG-D---SQQ---Y-------C-H-CCSE-C---VGH--MDCP
Ec-2  GC----------FDCATNICQF-----CE-AIVN-PNP--DM------WCKEA----QE---Y-------C-H-CCSE-C---VGH--MDCP
Ec-3  LC----------PGCAPNICQL-----CTYVVN–PNP--DVY-------CGDS----QE---Y-------C-H-CCSG-C---VGH--MDCP
Ef-α                  DDHCPTDVLMTC--GY-LQGRYNQGNYEEVGGLC---NM-SAEF----------C-H-CCSA-CDEPEVSPYSNCE
En-1                   NPEDWFTPDTC--AYG-D-SNT----AWT--TCTTP–G-------------QTC-YTCCSS-CFDVVG--EQACQMSA----Q-C
En-2                   DIEDFYTSETC--PYKND-SQL----AWD--TC--SGG-------T-----GNCGTVCCGQ-CFSFPVS--QSCAGMADSND--CPNA
En-A1                 YNPEDDYTPLTC--PHTISV-------VWYE--CT-------E----NTA---NCGTACCDS-CFELTG-NTM-CLLQAGAAGSGCDME
Ep-1                            SC---GS-------------E--CA------PE-------PD--C-WGCCLVQCA--PSI----CAG-------WCGGS
Ep-2                            SC---GS-------------E--CI------PE-------PD--C-WGCCLVQCD--PST----CVG-------WCGGS
```
